# Ectrodactyly/split hand feet malformation

**DOI:** 10.4103/0971-6866.60191

**Published:** 2009

**Authors:** Geetanjali Jindal, Veena R. Parmar, Vipul Kumar Gupta

**Affiliations:** Department of Pediatrics, Government Medical College, Sector 32, Chandigarh, India

**Keywords:** Autosomal recessive, split-hand/split-foot malformation, syndactyly

## Abstract

Split-hand/split-foot malformation is a rare limb malformation with median clefts of the hands and feet and aplasia/hypoplasia of the phalanges, metacarpals and metatarsals. When present as an isolated anomaly, it is usually inherited as an autosomal dominant form. We report a case of autosomal recessive inheritance and discuss the antenatal diagnosis, genetic counseling and treatment for the malformation.

## Introduction

Split-hand/split-foot malformation (SHFM)/ectrodactyly, also known as “lobster claw hand,” is a limb malformation involving the central rays of the autopod and presenting with syndactyly, median clefts of the hands and feet and aplasia/or hypoplasia of the phalanges, metacarpals and metatarsals. There is median cleft in the hand and feet due to the absence of the central digital rays, which gives the appearance of a lobster.[1]

We report a case with ectrodactyly involving both hands and feet.

## Case Report

**Figure 1a-d:**
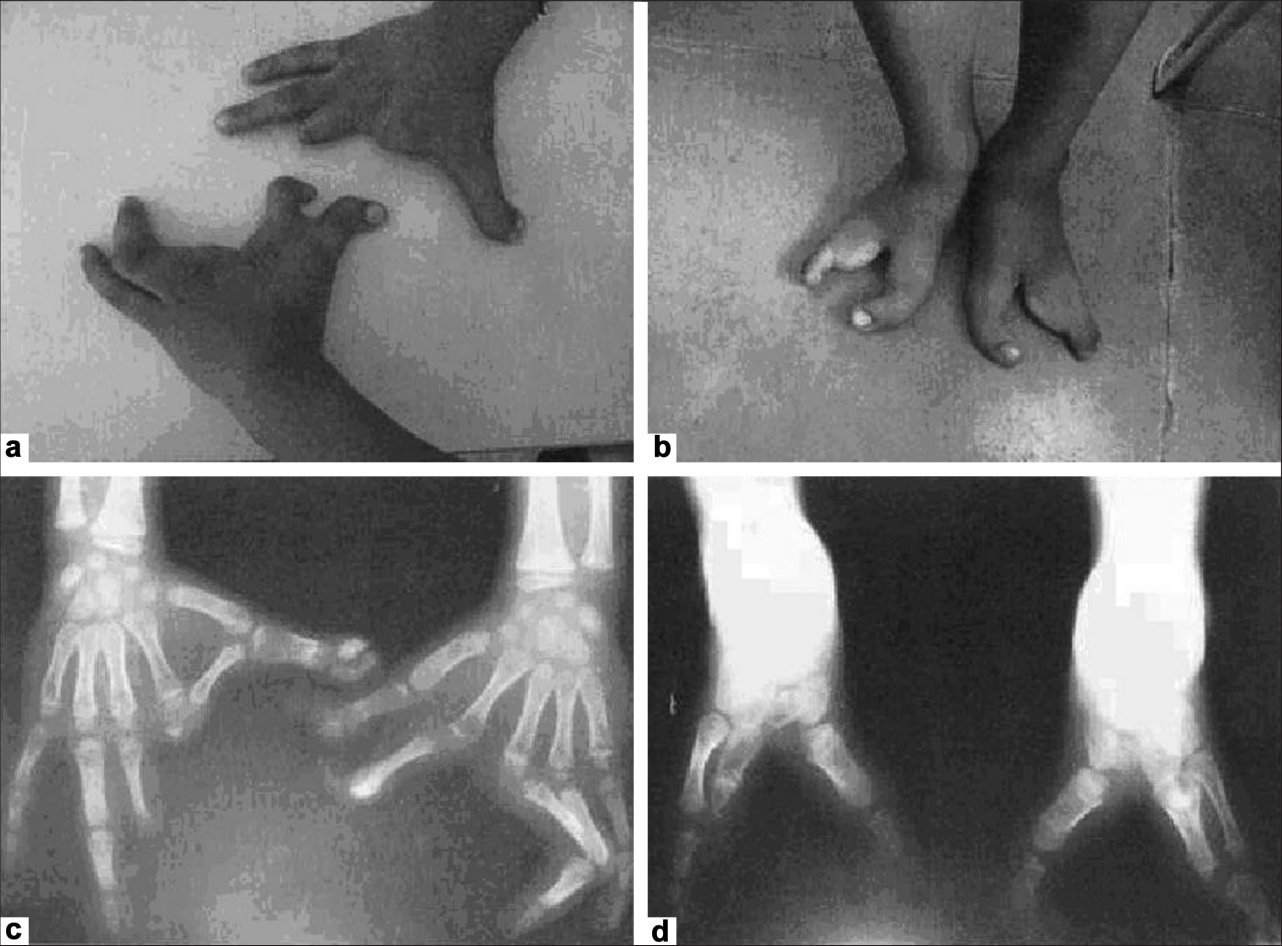
Split hand/split feet showing syndactyly, median clefts of the hands and feet and aplasia/or hypoplasia of the phalanges, metacarpals and metatarsals

A 6 ½-year-old boy presented with deformed hands and feet since birth. There were median clefts of both hands. In the left hand, there was syndactyly of the middle finger with ring finger and that of the thumb with the index finger and, in the right hand, there was syndactyly of the index finger and thumb [Figure 1a]. The X-rays of the hands [Figure 1b] showed normal metacarpals in both the hands but absence of middle and terminal phalanges of the middle finger in both hands. Both the feet also had a deep midline cleft [Figure 1c and d]. There was syndactyly with absence of multiple metatarsals and phalanges in both the feet.

There were no other dysmorphic features and anthropometry was within normal limits. Physical and systemic examination was normal. The child was a student of second standard, good in studies and had normal development for his age. He was a product of non-consanguineous marriage and term normal delivery with no significant perinatal events.

The index case was second in birth order. The first sibling was a female baby, still birth at term. This baby also had similar deformed hands and feet. Beside this abnormality, the parents did not notice any abnormal features. There was no history of similar clinical profile in any of the relatives of both the parents.

## Discussion

SHFM involves median clefts of the hands and feet with associated syndactyly, aplasia and/or hypoplasia of the phalanges, metacarpals and metatarsals.[1] Its incidence has been reported to be about 1 in 90,000 babies with no sex predeliction.[2] Two expressions of SHFM occur, one with isolated involvement of the limbs, known as the non-syndromic form, and the second, the syndromic form, with associated anamolies such as tibial aplasia, mental retardation, ectodermal and craniofacial findings, orofacial clefting and deafness.[3] Our case belongs to the non-syndromic type of SHFM as there is no associated anomaly.

Five different genetic mutations are known to be associated with SHFM. Type I, the most frequent variety, is due to a mutation on chromosome 7 in a region that contains two homeobox genes, DLX5 and DLX6.[1]

The syndromic form has a variable degree of expression. The non-syndromal SHFM limited to the hands and feet usually follows the pattern of inheritance of a regular autosomal dominant gene with a high penetrance.[4] However, in our case, the probable inheritance pattern is autosomal reccesive as only siblings and no other family member are affected.

There have been isolated case reports in the literature of the autosomal recessive inheritance pattern of SHFMs of the non-syndromal type. Verma *et al*. described split-hand and split-foot in two sibships born out of consanguineous marriage and indicated that split-hand and -foot deformity can be inherited as an autosomal recessive trait.[5] Ray and Freire-Maia also reported autosomal recessive ectrodactyly.[6,7] Klein also reported ectrodactyly in two siblings born out of mating between a man and the daughter of his half-brother.[8] Zlotogora and Nubani described a family is in which four subjects in two sibships had typical SHFM This also suggests an autosomal recessive form of the disorder. However, a two-locus model has also been suggested as an alternative possibility. In the two-locus model, the dominant mutation leading to the SHFM is controlled by a gene at another locus. A dominant mutation at the controlling locus leads to non-penetrance of the split hand/foot mutation and the appearance of normal carriers.[9]

Ectrodactyly can be treated surgically in order to improve function and appearance. Prosthetics may also be used.[2] Parents should be counseled regarding the possibility of recurrence of the disease in the future siblings and antenatal diagnosis by ultrasonography should be offered.[3,10]
